# Various Antibacterial Strategies Utilizing Titanium Dioxide Nanotubes Prepared via Electrochemical Anodization Biofabrication Method

**DOI:** 10.3390/biomimetics9070408

**Published:** 2024-07-05

**Authors:** Wuzhi Wang, Hanpeng Liu, Zilin Guo, Zijun Hu, Kefeng Wang, Yujia Leng, Caideng Yuan, Zhaoyang Li, Xiang Ge

**Affiliations:** 1 Key Laboratory of Mechanism Theory and Equipment Design of Ministry of Education, School of Mechanical Engineering, Tianjin University, Tianjin 300354, China; 2 Tianjin Key Laboratory of Composite and Functional Materials, School of Materials Science and Engineering, Tianjin University, Tianjin 300072, China; 3 School of Mechanical Engineering, Hebei University of Technology, Tianjin 300401, China; 4 National Engineering Research Center for Biomaterials, Sichuan University, Chengdu 610064, China; 5 School of Precision Instrument and Opto-Electronics Engineering, Tianjin University, Tianjin 300072, China; 6 School of Chemical Engineering and Technology, Tianjin University, Tianjin 300350, China

**Keywords:** titanium dioxide, electrochemical anodization, biofabrication method, orthopedic, implants, antibacterial properties

## Abstract

Currently, titanium and its alloys have emerged as the predominant metallic biomaterials for orthopedic implants. Nonetheless, the relatively high post-operative infection rate (2–5%) exacerbates patient discomfort and imposes significant economic costs on society. Hence, urgent measures are needed to enhance the antibacterial properties of titanium and titanium alloy implants. The titanium dioxide nanotube array (TNTA) is gaining increasing attention due to its topographical and photocatalytic antibacterial properties. Moreover, the pores within TNTA serve as excellent carriers for chemical ion doping and drug loading. The fabrication of TNTA on the surface of titanium and its alloys can be achieved through various methods. Studies have demonstrated that the electrochemical anodization method offers numerous significant advantages, such as simplicity, cost-effectiveness, and controllability. This review presents the development process of the electrochemical anodization method and its applications in synthesizing TNTA. Additionally, this article systematically discusses topographical, chemical, drug delivery, and combined antibacterial strategies. It is widely acknowledged that implants should possess a range of favorable biological characteristics. Clearly, addressing multiple needs with a single antibacterial strategy is challenging. Hence, this review proposes systematic research into combined antibacterial strategies to further mitigate post-operative infection risks and enhance implant success rates in the future.

## 1. Introduction

According to the “Medical Implants Market Size & Share Analysis–Growth Trends & Forecasts (2024–2029)” report by Mordor Intelligence, the global market size for medical implants is projected to reach USD 111.33 billion in 2023 and is anticipated to soar to USD 1576.2 billion by 2028, reflecting a compound annual growth rate of 7.2% [1]. The advancement of technology has facilitated more convenient travel for people, contributing to a rise in serious traffic accidents and a significant increase in orthopedic trauma among middle-aged and young individuals [2]. Additionally, the global aging population has led to a steady increase in orthopedic surgeries over the years [2]. Consequently, there is a growing demand for orthopedic implants, which currently hold the largest market share [3]. In the United States, the market value of orthopedic implants was USD 46.5 billion in 2018, and it is expected to climb to USD 64 billion by 2026 [4].

Currently, implant materials utilized in orthopedics are generally categorized into four groups: biometal, bioceramic, biopolymer, and biocarbon materials [5,6,7]. Biometal materials typically offer several benefits, including excellent mechanical properties, affordability, high corrosion resistance, favorable biocompatibility, effective bone integration, and adequate strength and toughness [8]. However, they are also susceptible to stress corrosion, brittleness, poor mechanical compatibility, loose prosthesis, and instability [9,10,11]. Bioceramics exhibit good biocompatibility, biological activity, wear resistance, and corrosion resistance, albeit with drawbacks such as poor mechanical compatibility and low ductility [12]. Biopolymers demonstrate good biocompatibility, corrosion resistance, and toughness, though their mechanical strength is comparatively weak, and their degradation products may alter the environmental pH [13]. Biocarbon materials boast excellent mechanical compatibility, biocompatibility, strength, and toughness, yet they exhibit poor biological activity [14]. Each of these materials presents its own set of advantages and disadvantages.

However, metals continue to be the predominant biomaterials utilized in clinical applications [15]. Over the past decades, metal biomaterials have stood as the gold standard in hard tissue applications, constituting approximately 70–80% of the manufactured implants [16]. In the late 1960s, titanium emerged as a pioneering implant material [17]. Subsequently, extensive research and numerous clinical cases worldwide have established titanium and titanium alloys as the foremost metal biomaterials for human implants, recognized for their comprehensive suitability [18,19,20,21]. Indeed, the advantages of titanium can be succinctly outlined as follows: lightweight, low elastic modulus, non-magnetic, non-toxic, excellent corrosion resistance, high strength, and superior toughness [2,21,22].

**Figure 1:**
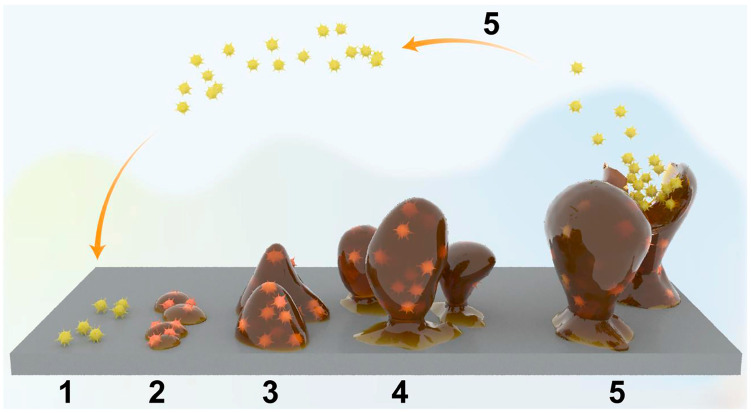
Schematic diagram of the five stages of mature biofilm formation by planktonic bacteria on an implant surface: (1–2) Adhesion of planktonic bacteria to an implant surface; (3) Biofilm formation on an implant surface; (4) Growth and maturation of biofilms; and (5) Dispersal of mature biofilms. Reproduced with permission from Ref. [23].

While titanium has emerged as the predominant implant material owing to its numerous advantages, bacterial infections of titanium-based materials frequently occur in clinical settings due to antibiotic overuse and the drug resistance of pathogenic bacteria [23,24,25,26,27]. Such infections can lead from impaired union to the amputation of affected extremities [28]. Indeed, post-operative infection stands as one of the most serious complications following implantation [29,30]. Not only does it jeopardize the success of implant surgery, but it also prolongs patients’ recovery time, imposing both mental and economic burdens [31,32]. Despite advancements in surgical techniques and aseptic practices in recent years, the average post-operative infection rate remains high, ranging from 2 to 5% [28,31]. Bacterial infections of implants primarily stem from bacterial adhesion and subsequent biofilm formation (Figure 1) on the material surface [29]. However, once a mature biofilm has formed on the implant surface, conventional treatment becomes challenging to eradicate it [33]. Consequently, inhibiting early bacterial adhesion before mature biofilm formation represents one of the most promising approaches [34], necessitating researchers to develop antibacterial surfaces on titanium implants.

**Figure 2:**
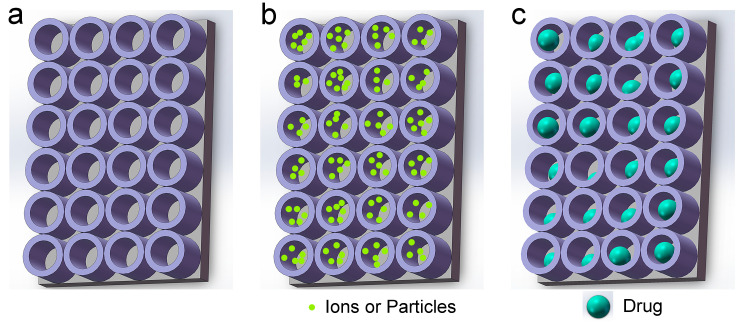
Schematic diagram of TNTA. (**a**) Pure TNTA; (**b**) Doped TNTA (Dopants are metal or non-metal ions, or particles); and (**c**) Drug-loaded TNTA (Drugs are antibacterial peptides (AMPs) or antibiotics).

In the quest to confer antibacterial properties onto titanium-based implant surfaces, the titanium dioxide nanotube array (TNTA) has garnered significant attention among researchers (Figure 2a). Several preparation methods for TNTA exist, including the template method, hydrothermal synthesis method, and electrochemical anodization method [35]. Among these, the electrochemical anodization method holds notable advantages, such as a safe preparation process, low pollution, and affordability. Of paramount importance is the ability to fabricate highly ordered TNTA through electrochemical anodization on the surface of titanium-based implants [36]. This method not only ensures good biocompatibility, but also holds potential antibacterial properties for implants [37,38]. For instance, the pore structure of the nanotubes on the surface can significantly influence bacterial activity and reproduction [39,40]. Furthermore, upon exposure to visible light or ultraviolet (UV) light, the surface of titanium dioxide can generate cytotoxic reactive oxygen species (ROS) [41,42], which exhibit bactericidal effects [43,44].

Furthermore, researchers can introduce metal or non-metal elements into TNTA to augment its antibacterial efficacy and cell compatibility (Figure 2b) [45,46]. Additionally, unlike solid structures, such as nano-column arrays or nano-needle arrays, TNTA on the surface of titanium substrates can be conceptualized as a hollow structure. Consequently, researchers can exploit this architecture to incorporate antibacterial drugs (for clarity, the drugs mentioned in this article encompass macromolecular antibacterial peptides (AMPs) and antibiotics) (Figure 2c) [47,48,49]. To achieve superior clinical outcomes, researchers have also integrated mechanical antibacterial methods, chemical antibacterial methods, and drug antibacterial methods to enhance the antibacterial efficacy and cell compatibility of titanium implants.

This review begins by presenting various preparation methods of TNTA, with a particular focus on elucidating the development process and the mechanism of the electrochemical anodization biofabrication method. Furthermore, we discuss the impact of different parameters on the preparation process. Subsequently, we introduce and summarize the latest advancements in various antibacterial strategies, encompassing topographical, chemical, drug delivery, and combined antibacterial strategies. Lastly, this review concludes by summarizing current findings and forecasting future research trends and development directions for titanium implants.

## 2. Preparation Methods of TNTA

### 2.1. Characteristics of Various Methods

Currently, commonly employed preparation methods for TNTA encompass the template synthesis method, sol-gel method, hydrothermal method, and electrochemical anodization method [50,51]. Table 1 presents the advantages and disadvantages of each method.

The template synthesis method entails the use of templates to assist in the preparation process [52]. Here, the precursor is deposited into a hole with specific dimensions or onto a surface through physical or chemical means [52]. Subsequently, the template is removed, resulting in the formation of a nanotube array with defined shape, structure, and size [52].

The sol-gel method represents a chemical approach utilized for synthesizing various nanostructures [53]. Typically, the sol-gel method comprises five fundamental steps: hydrolysis, polycondensation, aging, drying, and thermal decomposition [54]. During hydrolysis, precursors undergo hydrolysis in water or alcohol [54]. The subsequent step, polycondensation, involves the condensation of adjacent molecules [54]. This process leads to an increase in solvent viscosity, resulting in the formation of a porous structure termed gel, while the solution remains in a liquid phase [54]. Aging is aimed at continually altering the structure and properties of the gel, ultimately reducing porosity and increasing interparticle thickness [54]. The drying phase involves the separation of water and organic matter [54]. Finally, thermal treatment is conducted to eliminate residues and water molecules from the sample [54]. The temperature of the thermal treatment is a critical parameter for controlling pore size and material density [54].

The hydrothermal method is commonly employed for synthesizing nano-powders. However, when utilized for preparing nanotubes, the resulting nanotubes exhibit poor thermal stability, rendering them thin and susceptible to environmental factors. Consequently, accurate analysis of the nanotube structure and composition becomes challenging [35]. The crux of the hydrothermal method lies in utilizing an aqueous solution as the reaction solvent and employing a stainless-steel reactor capable of withstanding high temperatures and pressures as the reaction vessel.

The electrochemical anodization method involves anodizing titanium in an electrolyte containing fluoride [21,55]. This method is relatively simple yet highly effective, allowing for the formation of highly ordered TNTA [21,55]. Given its capability to directly fabricate nanostructures on titanium or titanium alloys, this review primarily concentrates on elucidating the development history and manufacturing mechanism of the electrochemical anodization method.

**Table 1:** The characteristics of different preparation methods for TNTA.

Methods	Advantages	Disadvantages	Ref.
Template synthesis method	1. Controlled scale of nanotubes using various templates 2. More desirable for practical applications	1. Long-term instability 2. Complex manufacturing process	[35,52]
Sol-gel method	1. Simple process 2. High production efficiency 3. Low initial investment with high-quality products	Long manufacturing duration	[53]
Hydrothermal method	1. Efficient route for large-scale production 2. High length-to-diameter ratio	1. Long reaction duration 2. Thermally unstable	[35]
Electrochemical anodization method	1. Simplicity 2. Cost efficiency 3. Exhibiting exceptional electrical, optical, structural, and thermal properties	1. Constrained mass production 2. The utilization of highly toxic solvent	[21,55]

### 2.2. Development History of the Electrochemical Anodization Method

**Figure 3:**
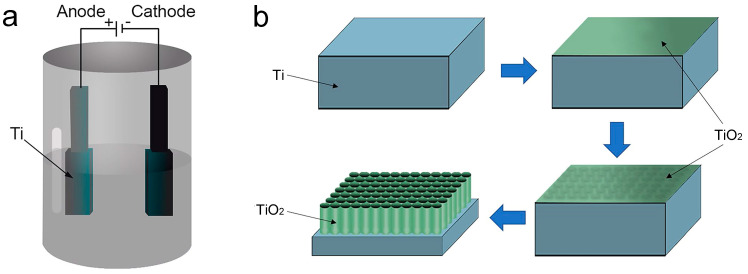
The growth process of TNTA is depicted in a schematic diagram utilizing a two-current model. (**a**) Electrochemical anodization reaction device; and (**b**) TNTA construction process. Reproduced with permission from Ref. [47].

The electrochemical anodization method typically employs a two-electrode system for the preparation of TNTA, wherein the anode comprises a titanium-based material and the cathode is either a platinum or graphite electrode (Figure 3a,b) [56,57,58]. The electrolyte commonly consists of either an aqueous solution or an organic solvent containing fluoride ions [59,60]. However, fluoride-free electrolytes have also been identified as viable alternatives [61,62]. During operation, the application of a specific voltage or current between the two electrodes leads to the formation of an oxide film on the anode [55]. The classification of electrochemical anodization methods primarily relies on the composition of the electrolyte [55]. Electrolytes used for preparing TNTA through the electrochemical anodization method can be broadly categorized into hydrofluoric (HF) acid aqueous solution electrolytes, fluoride aqueous solution electrolytes, fluorinated organic solution electrolytes, and fluoride-free electrolytes based on the main solvent [55,57].

#### 2.2.1. Preparation of TNTA in HF Aqueous Solution

In 2001, Gong et al. achieved the successful preparation of highly ordered and closely aligned TNTA on a titanium substrate in an aqueous solution containing 0.5~3.5% HF using the electrochemical anodization method, marking the first instance of such fabrication [56]. The diameter of the titanium dioxide nanotube (TNT) ranges from 25 to 65 nm, with the TNT diameter increasing with higher anodizing voltages [56].

Subsequently, numerous researchers delved into studying the fabrication methods, mechanisms, and applications of one-dimensional titanium dioxide [63,64,65,66]. Notably, due to the high oxide dissolution rate, HF exhibits robust corrosion capabilities towards TNT in this system [55]. Dissolution occurs rapidly at the openings of formed TNT, eventually reaching a balance between dissolution and production rates within a short timeframe [67]. Consequently, the length of TNT generated by these methods typically remains less than or equal to 500 nm [55]. Additionally, researchers observed a “rib”-like connection between these nanotubes [56].

However, the short length, poor self-organization, and rough surface of TNTA mentioned above hinders its widespread application.

#### 2.2.2. Preparation of TNTA in Fluoride Aqueous Solution

To address the limitations posed by the first-generation manufacturing methods, researchers turned their focus towards developing second-generation electrolytes. By substituting fluoride for HF in the electrolyte and adjusting its pH value, researchers successfully mitigated the corrosion of fluoride-containing electrolytes on formed TNT, thereby reducing the dissolution rate at the TNT openings. Moreover, modifications to reaction parameters such as oxidation time enabled the attainment of TNT lengths extending to several microns [67,68,69,70].

Lockman et al. introduced Na_2_SO_4_ into the bulk electrolyte composed of 5 wt.% NH_4_F and subsequently adjusted the pH value to 3 by adding H_2_SO_4_ [58]. Using this modified electrolyte, self-organized TNTAs were fabricated by anodizing pure titanium foil in a standard double-electrode bath [58]. Similarly, Macak et al. investigated the anodic formation of TNTA on titanium in Na_2_SO_4_ electrolytes containing NaF (0.1–1 wt.%) [69]. The resulting TNTA typically exhibited a single pore size of 100 nm and an average spacing of 150 nm [69].

#### 2.2.3. Preparation of TNTA in Fluorinated Organic Solution

As research has advanced, it has become evident that the properties of the electrolyte significantly influence the morphology of TNTAs prepared via the electrochemical anodization method. Particularly, when the primary constituent of the electrolyte is an organic solvent, the length of TNTs can be substantially increased. This enhancement is attributed to the elevated viscosity of the solution and the consequent reduction in the diffusion rate of fluoride ions [71].

This discovery has facilitated the fabrication of TNTAs featuring regular nozzle shapes, uniform TNT diameters, smooth TNT walls, and high aspect ratios using the electrochemical anodization method. Such breakthroughs have effectively addressed the application limitations stemming from inadequate TNT length and other challenges encountered during the early stages of development. Consequently, new possibilities have been unveiled for the utilization of TNTAs across various fields.

Macak et al. achieved a TNTA with a length of 7 μm in a glycerol solution containing NH_4_F (mass fraction 0.5%) [68]. The resulting TNTs exhibited excellent self-organization, smooth TNT walls, and absence of “ribs”, marking a significant advancement in the preparation of self-organized and orderly TNTA [68]. Additionally, Prakasam et al. discovered that the growth rate of TNT in fluoride-containing organic solution electrolytes can reach as high as 15 μm/h, surpassing rates observed in other electrolytes by 7.5~60 times [72]. The research team continuously set new records, producing TNTA films with TNT lengths of 134 μm [73], 220 μm [74], 360 μm [72], 720 μm [75], and 1000 μm [76].

Subsequently, Jaroenworaluck et al. achieved the hexagonally close-packed TNTA with improved orderliness in a glycol solution containing NaF (0.3 wt.%) and Na_2_SO_4_ (1 M) [77]. This led to a faster electrochemical anodization rate, enabling TNT lengths to reach tens of microns [77].

In 2009, Wang et al. proposed a two-step anodization process for TNTA preparation [78]. Initially, conventional electrochemical anodization was employed, followed by the removal of the first oxide layer [78]. The remaining high-purity titanium sheet surface retained bowl-shaped corrosion pits, serving as the initial oxide layer for the second anodization [78]. This process yielded a TNTA thin film exhibiting nanoscale surface flatness characteristics [78]. Additionally, Shin et al. combined electrolytic polishing of Ti with the two-step electrochemical anodization method to achieve an almost perfectly regular hexagonal TNTA structure [79].

#### 2.2.4. Preparation of TNTA in Fluorine-Free Solution

An increasing number of studies have demonstrated the feasibility of obtaining TNTA in fluoride-free electrolytes. In 2007, Hahn et al. achieved the TNTA using the electrochemical anodization method in an electrolyte containing perchloric acid (HClO_4_) [80]. The resulting TNTs exhibited a diameter of approximately 40 nm, a wall thickness of about 10 nm, and a length of approximately 30 μm [80]. Additionally, Richter et al. investigated TNTA fabrication in different electrolyte systems comprising 0.4 mol/L NH_4_Cl + 0.5 mol/L oxalic acid, 0.4 mol/L NH_4_Cl + 0.5 mol/L formic acid, and 0.4 mol/L NH_4_Cl + 0.05 mol/L sulfuric acid [81]. The synthesized TNT had a diameter of about 20 nm and a length ranging from 5 to 50 μm [81]. However, it was observed that while TNTs were obtained in fluoride-free electrolytes within a short duration, their arrangement was uneven, and the film could detach from the titanium substrate during the preparation process [80].

A common feature among these experiments is the presence of chloride ions or perchloric acid in the electrolytes, despite the absence of fluoride. Subsequently, in 2009, Fahim et al. demonstrated that TNTA could be prepared in an electrolyte containing sulfuric acid without the addition of other acids under an appropriate electrochemical anodization voltage [61].

**Figure 4:**
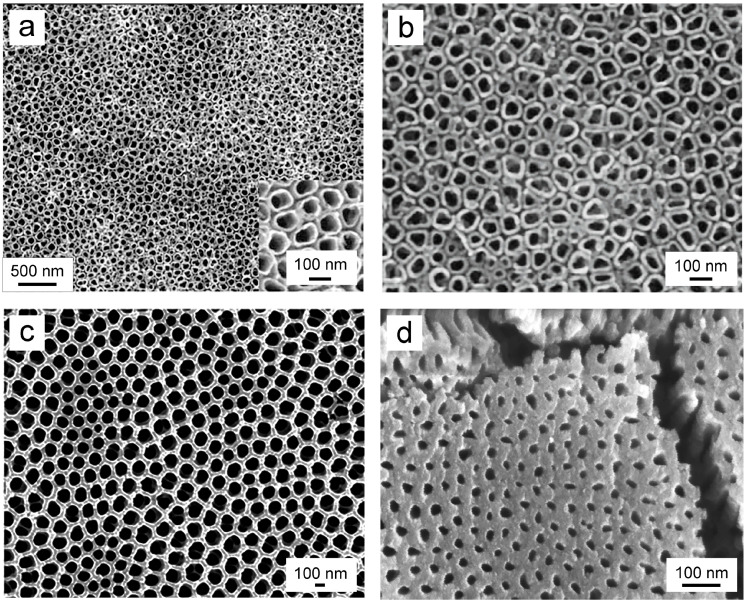
SEM images of the TNTAs prepared in four different types of electrolytes. (**a**) The TNTA prepared in an HF aqueous solution [82]; (**b**) The TNTA prepared in a fluoride aqueous solution [83]; (**c**) The TNTA prepared in a fluoride organic solution [84]; and (**d**) The TNTA prepared in a fluorine-free electrolyte [85]. Reproduced with permission from Refs. [82,83,84,85].

Researchers currently utilize electrolytes for preparing TNTA, classified into the four categories mentioned earlier. The selection and development of electrolytes are summarized in Table 2. SEM images of TNTs prepared with different electrolytes are depicted in Figure 4a–d. Among these categories, fluorine-containing organic solution electrolytes offer notable advantages. They are relatively safe, environmentally friendly, and promote rapid and uniform growth of TNTA. Consequently, fluorine-containing organic solution electrolytes have become the preferred choice for preparing TNTAs via the electrochemical anodization method.

**Table 2:** The development process of the electrolytes.

Electrolyte Type	Electrolyte Composition	U/V	t/h	D/nm	L/nm	Year	Ref.
Aqueous Solution	0.5 wt.% HF	20	1/3	60	250	2001	[56]
	H_2_SO_4_ + HF	20	24	140	580	2003	[63]
	1 M (NH_4_)_2_SO_4_ + 0.5 wt.% NH_4_F	20	2	90–110	2000	2005	[86]
	1% HF + 2.5% HNO_3_	20	4	100	400	2006	[87]
	1% HF + 2.5% HNO_3_ + 0.5 M H_3_BO_3_	20	4	120	560	2006	[87]
	0.1 M KF + 1.0 M NaHSO_4_	20	1	110	1800	2007	[60]
	1 M Na_2_SO_4_ + 0.5 wt.% NaF	20	2	100	500	2013	[59]
Organic Solution	0.5% HF + Acetic Acid	10	N/A	22	224	2005	[88]
	0.25% NH_4_F + Ethylene Glycol	60	1	80	12,000	2007	[60]
	0.56 g NH_4_F + 5 mL Deionized Water + 95 mL Formamide	20	54	N/A	28,000	2007	[74]
	0.56 g NH_4_F + 5 mL Deionized Water + 95 mL Formamide	35	80	N/A	58,000–66,000	2007	[74]
	Dimethyl sulfoxide electrolyte containing 2 vol.% HF	60	70	150	93,000	2007	[74]
Fluorine-Free Solution	0.5 M Oxalic Acid + 0.1 M KCl + 0.15 M NH_4_Cl + 0.15 M KOH	18	N/A	20	5000–50,000	2007	[81]
	0.5 M Oxalic Acid + 0.3 M NH_4_Cl	13	N/A	N/A	60,000	2007	[81]
	0.05 M HCl	10	1	10	/	2007	[62]

Definition of the letters: U: voltage; t: duration; D: diameter; L: length; Year: publication year.

### 2.3. Mechanism of the Electrochemical Anodization Method

The electrochemical anodization method employed for processing titanium surfaces operates under the influence of an electric field. This field facilitates the diffusion of metal ions and oxygen ions, leading to the formation of oxide films at two distinct interfaces: the titanium/titanium oxide interface and the titanium oxide/electrolyte interface, respectively [55].

The oxide film formed at the interface of titanium and titanium oxide is determined by the migration of Ti^4+^ ions released during metal oxidation through the oxide film to the titanium oxide/electrolyte interface, along with the combination of O^2−^ and OH^−^ ions [55]. Conversely, the oxide film at the titanium oxide/electrolyte interface is established through the migration of O^2−^ and OH^−^ anions from the aqueous solution to the metal/oxide interface, where they combine with Ti^4+^ ions [55].

During the electrochemical anodization process, the overall reaction can be represented as follows [55]:

Ti^2+^ + 2O^2−^ → TiO_2_ + 2e^−^ (1)

At the titanium/titanium oxide interface, the reaction can be described as follows [55]:

Ti → Ti^2+^ + 2e^−^ (2)

At the titanium oxide/electrolyte interface, the reactions can be represented as follows [55]:

2H_2_O → 2O^2−^ + 4H^+^ (3)

2H_2_O → O_2_ + 4H^+^ + 4e^−^ (4)

**Figure 5:**
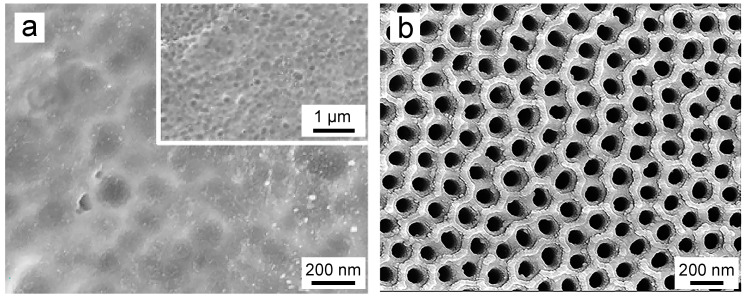
SEM images of titanium dioxide on the titanium surface: (**a**) Dense type [90]; and (**b**) Porous type [92]. Reproduced with permission from Refs. [90,92].

The formation of oxide films during the electrochemical anodization process is influenced by various factors, including the composition of the electrolyte, duration of oxidation, applied voltage, and temperature [89]. It is widely accepted that the application of acidic or saline solutions yields a dense anodic oxide film (Figure 5a) [55,90]. Conversely, the presence of fluoride ions in the electrolyte leads to the formation of a porous anodic oxide film, specifically TNTAs (Figure 5b) [57,91,92]. Additionally, the solubility of titanium dioxide in the electrolyte dictates the film’s morphology: insolubility results in a dense film, while solubility facilitates the formation of TNTAs [57,91].

The electrochemical anodization current density-time curves corresponding to the two types of oxide films exhibit distinct characteristics. The curve of the dense anodic oxide film can be divided into two stages. In the first stage, the oxide film thickness gradually increases during the electrochemical anodization process, and the total resistance becomes larger, thus impeding the migration of ions [93,94]. In the second stage, once the oxide film thickness reaches a certain level, the ions no longer migrate, the current density remains stable, and the oxide film growth ceases [93,94].

The curve representing TNTA can be segmented into three distinct parts [94,95]. Firstly, during the initial stage, the electric field within the oxide film is intense, resulting in a substantial ionic current [94]. This rapid increase in current density leads to the rapid growth of the anodic titanium oxide film, causing an exponential decrease in the overall current density [94]. Concurrently, under the influence of the electric field, oxides grow simultaneously at both the titanium/titanium oxide interface and the titanium oxide–electrolyte interface, forming dense anodic oxide [94]. Secondly, as the thickness of the anodic oxide film reaches a critical point, the primary mode of conduction transitions from ionic to electronic conduction, leading to an increase in the sum of the two currents [94]. This shift occurs due to the embedding of anions such as F^−^/O^2−^ and OH^−^ in the electrolyte during the oxidation process of the anodic oxide film, resulting in the formation of a pollution layer near the electrolyte interface [96,97]. As the thickness of the pollution layer increases, anions accumulate at the pollution layer/barrier interface [98]. This accumulation enhances the likelihood of anions separating oxygen under the influence of the electric field and electronic current [98]. Consequently, due to the obstruction of the pollution layer, oxygen bubbles form the initial nuclei for pores within the film [99]. The extent of anion intercalation determines whether a porous or dense oxide film is formed [100]. Thirdly, TNTs emerge on the pollution layer and barrier layer. As the electrolyte penetrates, the thickness of the barrier layer at the bottom of the TNTs continues to evolve until it reaches a critical thickness [101,102,103]. Throughout this phase, the two currents remain unchanged, and the total current remains stable [101,102,103]. Notably, the electric field is most intense at the bottom of the TNT, leading to upward growth of the oxide around the bubble at the bottom of the TNT, ultimately resulting in gaps between the TNTs [101,102,103].

### 2.4. Effects of Different Parameters of Electrochemical Anodization

During the preparation process, various parameters critically influence the final morphology of TNT, such as electrolyte composition, anodization voltage, anodization time, and anodization temperature [55].

Indeed, among all the parameters, the electrolyte composition exerts the most significant influence on the final morphology of TNT. This perspective is supported by SEM images corresponding to the four different electrolytes mentioned above (Figure 4a–d). The viscosity of different electrolytes varies, thereby affecting the mobility and conductivity of ions in the electrolyte [55]. Typically, TNTs grown in organic electrolytes tend to be longer and smoother compared to those grown in aqueous electrolytes [104]. Presently, the most commonly utilized organic electrolytes are prepared based on glycerol and glycol solutions [104].

Anodizing voltage plays a significant role in the formation of TNTs [105]. Low voltages result in the formation of a mixed structure between the top nanopore layer and the bottom TNT layer [106], whereas high voltages induce the rapid dissolution of titanium dioxide, leading to the attenuation or fracture of TNTs, and in severe cases, the fracture of the titanium substrate [93]. Therefore, the preparation of TNTs via the electrochemical anodization method requires careful control within a specific voltage range [93]. Furthermore, the diameter and length of TNTs increase with an increase in oxidation voltage, with the degree of linearity dependent on the oxidation voltage [107]. However, exceeding the critical voltage value may lead to a decrease in the diameter of TNTs [104].

The anodizing time primarily influences the length of TNTs [93]. Studies have shown that as the anodizing time increases, the length of the nanotubes grows longer, and the uniformity of pore distribution in nanostructures improves [104]. However, this effect is not unlimited [104]. Lengthening the anodizing time during the initial stages enhances the length of TNTs [104]. Once the growth rate of TNTs matches the chemical etching rate, the TNT length stabilizes and no longer increases [104].

Anodizing temperature is indeed a crucial parameter in the preparation process. Research indicates that the wall thickness of TNT can be regulated by varying the electrochemical anodization temperature [108]. In 2005, Mor et al. reported that the wall thickness of TNT increases as the anodizing temperature decreases, while the length of TNT increases with a decrease in the anodizing temperature [88]. Additionally, Wang et al. observed that the diameter of TNT prepared via the electrochemical anodization method in an ice bath is even smaller [109]. This highlights the significant impact of anodizing temperature on the morphology and dimensions of TNT.

## 3. Antibacterial Strategies

The advent of biomedical implants has revolutionized medicine, profoundly impacting the lives of numerous individuals worldwide. In the United States alone, implant-related infections contribute to tens of thousands of fatalities annually and incur billions of dollars in hospital costs [110]. While implants hold the potential to save lives and enhance quality of life, the associated risk of infection cannot be overlooked. For instance, in orthopedic surgery, the current global infection risk stands at 2–5% [37]. Hence, it is evident that infection presents a prevalent and intricate challenge following implant surgery. Recently, considerable attention has been directed towards imbuing implant surfaces with effective antibacterial properties, representing a focal point of research for scientists and clinical experts [23]. Given the various biological behaviors exhibited by bacteria, particularly in forming biofilms post-infection, two primary types of surfaces have been developed to impede bacterial adhesion and biofilm formation: bacteria-repelling surfaces and bacteria-killing surfaces [111]. Moreover, specific antibacterial strategies can be categorized into four main types: topographical, chemical, drug delivery, and combined antibacterial strategy.

### 3.1. Topographical Antibacterial Strategy

The comprehension of the interaction between micro/nanostructured surfaces of implants and bacteria can be enhanced through calculation. For instance, consider a cube with a side length of 1 cm: its macroscopic surface area measures 6 cm^2^ [40]. However, upon dividing it into cubes with a side length of 1 mm, the surface area escalates significantly to 60 cm^2^ [40]. Further subdivision into cubes with a length of 1 nm results in an astonishing increase to 60,000,000 cm^2^ [40]. Hence, implants featuring micro/nanostructures can markedly amplify the specific surface area. Moreover, manipulation of the micro/nanostructured surface morphology of implants can effectively diminish bacterial adhesion on the titanium surface, while concurrently disrupting the bacterial cell membrane structure through the topological features of the implant surface [112,113].

**Figure 6:**
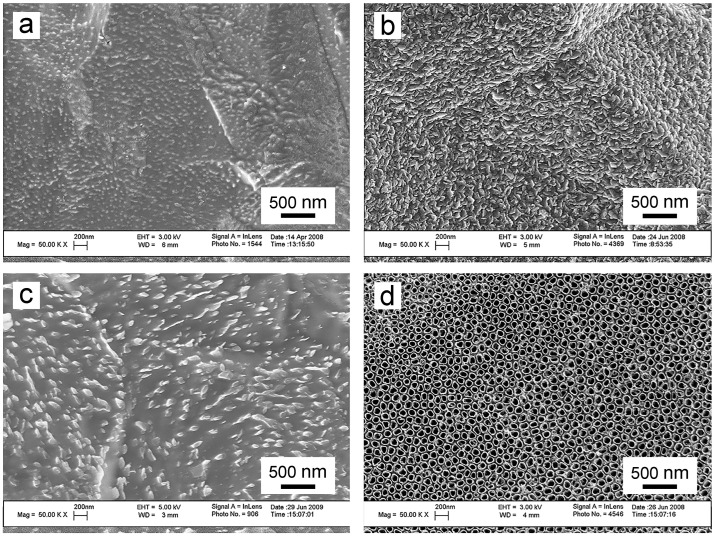
SEM images depicting Ti before and after electron beam evaporation and electrochemical anodization. (**a**) Conventional Ti as purchased from the vendor; (**b**) Nanorough Ti after electron beam evaporation; (**c**) Nanotextured Ti after electrochemical anodization for 1 min in 0.5% HF at 20 V; and (**d**) Nanotubular Ti after electrochemical anodization for 10 min in 1.5% HF at 20 V. Reproduced with permission from Ref. [114].

In 2009, Puckett et al. conducted a study examining the adhesion behavior of *Staphylococcus aureus* (*S. aureus*), *Pseudomonas aeruginosa* (*P. aeruginosa*), and *Staphylococcus epidermidis* (*S. epidermidis*) on traditional titanium surfaces compared to those produced via electron beam evaporation and electrochemical anodization techniques (Figure 6a–d) [114]. Their findings revealed that nanorough titanium surfaces (Figure 6b) generated by electron beam evaporation exhibited reduced bacterial adhesion, whereas nanotextured (Figure 6c) and nanotubular (Figure 6d) titanium surfaces created through electrochemical anodization demonstrated increased bacterial adhesion relative to smooth titanium surfaces [114].

**Figure 7:**
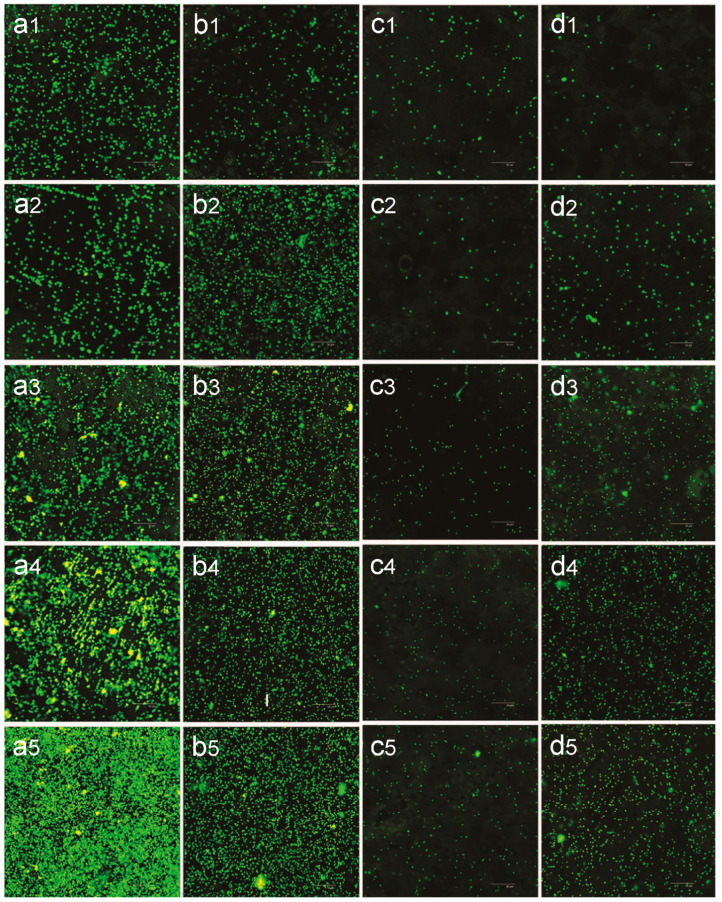
Confocal laser scanning microscopy (CLSM) analysis of *S. epidermidis* colonies on various surfaces. (**a**) Mechanically polished titanium; (**b**) Acid-etched titanium; (**c**) 80 nm TiO_2_ nanotube arrays; and (**d**) 30 nm TiO_2_ nanotube arrays. Each panel includes subpanels labeled (**1**) to (**5**), corresponding to different time points of incubation: 1 h, 2 h, 3 h, 4 h, and 5 h. The images were captured at 400× magnification. Reproduced with permission from Ref. [115].

In 2013, Peng et al. fabricated TNTs on titanium plates through a two-step electrochemical anodization process [115]. The tube diameters of the samples were 30 nm and 80 nm, respectively [115]. By employing mechanically polished titanium samples and corroded titanium samples as control groups, they observed a significant reduction in initial adhesion and colonization of *S. epidermidis* on the surface of TNTA (Figure 7a1–d5), while the adhesion of C_3_H_10_T1/2 cells (a type of mouse embryonic fibroblast) on TNT surfaces was notably enhanced [115]. Analyzing the roughness, water contact angle data, and antibacterial experimental results of four sample groups (Figure 7), the researchers indicated that when the nanotube diameter of TNTAs was 80 nm, it exhibited superior antibacterial properties against various *S. aureus* strains attributed to its higher hydrophobicity [115].

In 2021, Ji et al. investigated the antibacterial mechanism of TNTAs during various growth stages of *Escherichia coli* (*E. coli*) [24]. Ji observed a significant early antibacterial effect of TNTAs, with this effect strongly correlated with surface free energy and nano morphology [24]. Additionally, TNTs with larger diameters exhibited greater long-term antibacterial potential [24].

In a separate study, Ercan et al. subjected TNTs with varying diameters (20, 40, 60, and 80 nm) to heat treatment, and assessed their adhesion to gold and *E. coli* [116]. They determined that among all samples, the 80 nm TNT sample after heat treatment demonstrated the most effective antibacterial properties [116]. Furthermore, the antibacterial efficacy of TNTAs could be modulated by altering parameters such as heat treatment conditions (e.g., holding temperature and duration) and TNT diameter [116].

Presently, researchers have not arrived at a consensus regarding the relationship between the diameter of TNTs and their antibacterial efficacy. While Tang et al. [115] and Simi et al. [117] have corroborated that the bacterial inhibition rate of TNTAs increases with the rise in TNT diameter, contrasting conclusions have been drawn by other researchers. Lewandowska et al. demonstrated that TNTAs prepared at 4 V exhibit stronger antibacterial activity against various strains of *S. aureus* (with different samples subjected to voltages of 4, 6, 8, and 18 V, respectively) [118]. Similarly, Radtke et al. reported that, compared to other TNTAs, those with small diameters prepared at 5 V exhibit superior antibacterial effects [119]. Upon analysis of these findings, it is speculated that the discord among different research groups may arise from challenges in accurately controlling the geometric dimensions of TNTAs across various experiments. Besides TNT diameter, numerous factors could influence TNTA morphology, including TNT length, gap between TNTs, TNT wall thickness, and the crystal phase of TNT. Hence, accurately isolating the effect of TNT diameter on antibacterial properties becomes challenging amidst the variability in these other parameters. This article argues that without modifying the structure of TNTA, it is challenging to completely overcome the disadvantage of poor topographical antibacterial effect solely by altering the pore size, length, and other dimensions. Therefore, future research should focus on combining TNTA with additional antibacterial strategies, allowing topographical antibacterial properties to play a complementary role.

### 3.2. Chemical Antibacterial Strategy

Since the inception of combining antibacterial metals with TNTs, various metals, non-metal ions, or other nanoparticles have been extensively researched and applied to TNTs [45,46,120]. As research progresses, a clearer understanding of the specific role of ions has emerged. Their functions can be categorized as follows: (1) the ion itself possesses inherent antibacterial properties; (2) the ion can decrease the optical band gap value of TiO_2_, thereby enhancing the photocatalytic and antibacterial activity of TiO_2_ photocatalyst [93,121]. Indeed, both metal and nonmetal doping can reduce the band gap of TiO_2_ or facilitate in-band transmission, consequently reducing the light energy required for photoactivation [122]. Moreover, some studies have demonstrated that metal nanoparticles can effectively eradicate drug-resistant bacteria, particularly in scenarios where bacteria exhibit resistance to conventional drugs [123]. Hence, it is believed that chemical antibacterial strategies hold significant promise for future applications.

**Figure 8:**
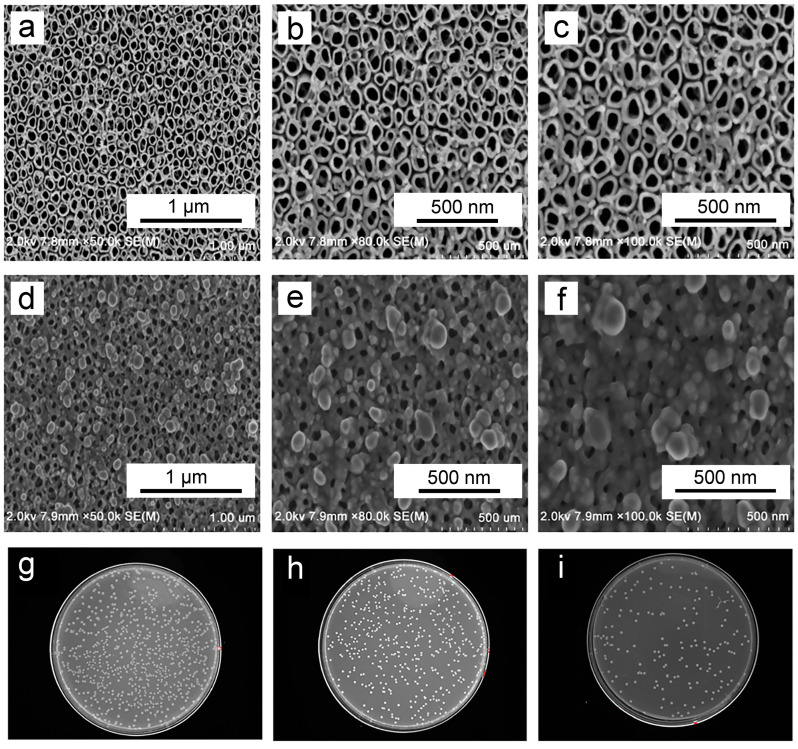
(**a**–**c**) Surface morphology of TNTs at varying magnifications; and (**d**–**f**) Silver-doped TNT (Ag-TNT) at varying magnifications. Additionally, *S. aureus* adherence to titanium (**g**), TNTs (**h**), and Ag-TNTs (**i**) is shown. Reproduced with permission from Ref. [127].

Silver (Ag) ions and Ag particles are the most commonly utilized dopants due to their ability to achieve optimal antibacterial effects while minimizing cytotoxicity [124,125,126]. Li et al. conducted electrochemical anodization of titanium plates in AgNO_3_ solution under xenon light irradiation [127]. Comparative analysis with pure TNTAs (Figure 8a–c) revealed successful Ag doping onto TNTAs, with nanoparticles predominantly distributed on the TNTA surface (Figure 8d–f) [127]. Antibacterial experiments further demonstrated that Ag-coated TNTs on titanium implants exhibit excellent antibacterial activity (Figure 8g–i) [127]. Similarly, Durdu et al. fabricated TNTAs via electrochemical anodization on titanium surfaces and subsequently deposited Ag nanoparticles randomly on the TNTAs using the electrodeposition process [128]. Experimental results demonstrate that these samples exhibit enhanced corrosion resistance and improved cell compatibility [128]. Notably, they also display significant antibacterial properties against *E. coli* and *S. aureus* [128]. Collectively, these experiments underscore the promising potential of Ag doping in enhancing the antibacterial efficacy of titanium implants.

**Figure 9:**
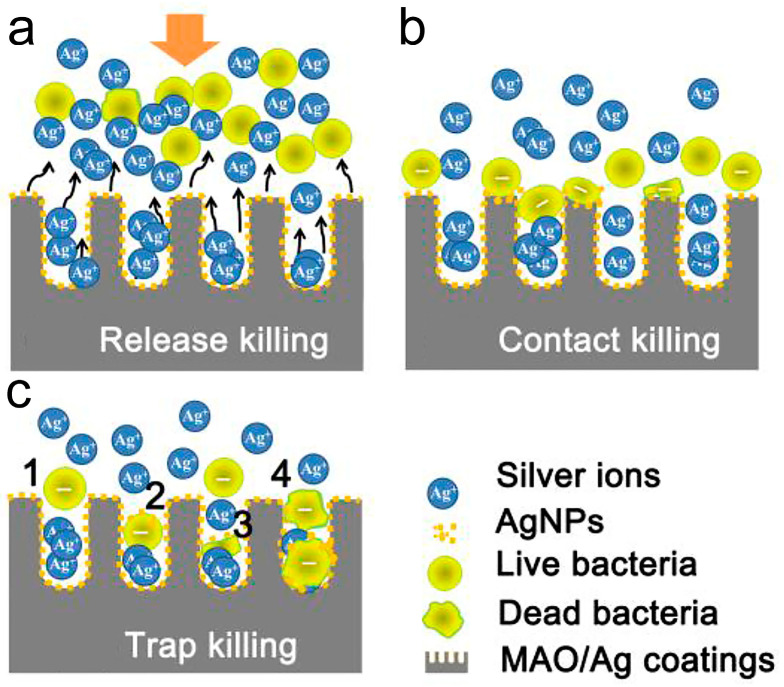
Schematic illustration of the possible killing modes involved during adhesion stage: (**a**) The majority of planktonic bacteria were repulsed from the surface by the releasing Ag^+^; (**b**) Some of the landed bacteria were disrupted via contact with AgNPs on the surface; and (**c**) Other survivors with negative membrane charge were attracted into the micropores (positively-charged by interior silver) and were killed by the so-called “trap-killing” principle. This principle may involve the following procedures: (1) Bacterial falling and colliding with pore walls; (2) Nanosilver binding through electrostatic attraction or specific interactions such as Ag-thiol bonds; (3,4) Membrane distortion and destruction by nanosilver and localized Ag^+^ for one or more bacteria. Reproduced with permission from Ref. [129].

Research indicates that the antibacterial mechanism of Ag in implants primarily comprises (Figure 9a–c): (1) repulsion of the majority of planktonic bacteria from the surface by released Ag^+^ ions; (2) disruption of some surface-landed bacteria through direct contact with Ag; (3) attraction and elimination of other surviving bacteria with negatively charged membranes into micropores, which are positively charged by interior Ag, based on the “trap-killing“ mechanism [129].

In addition to Ag, researchers have also conducted antibacterial experiments using other metal dopants such as copper (Cu) and zinc (Zn). Xie et al. employed a combination of electrochemical anodization, sandblasting, and acid etching to fabricate a composite film comprising titanium and copper oxide [130]. Their findings demonstrated that the surface-modified sample exhibited robust long-term antibacterial efficacy, with an antibacterial rate exceeding 99% [130]. Moreover, results from cell compatibility assessments indicated that the modified sample exhibited favorable cell compatibility [130]. Furthermore, researchers have substantiated that Cu not only enhances surface osteogenesis and antibacterial activity, but also promotes angiogenesis [45]. Despite Cu’s ability to promote osteogenesis and angiogenesis, its antibacterial efficacy falls short of that exhibited by Ag [45].

In terms of nonmetal dopants, fluorine (F), phosphorus (P), and other elements are commonly used. Conde et al. prepared F-containing TiO_2_ films by electrochemical anodization and conducted experiments to verify that the multifunctional surfaces produced by the electrochemical anodization process not only exhibited improved antibacterial properties but also possessed osteogenic properties beneficial for the fixation of prosthetic devices onto bone [131]. Ge et al. further indicated that fluoride ions, when used as an antibacterial agent, can significantly impact bacterial metabolism [132].

Liu et al. doped nitrogen (N) into electrochemically anodized TNTs by ion implantation [133]. It was found that N ion implantation improved the corrosion resistance of TNTs and enhanced their hydrophilicity [133]. The antibacterial activity of ion-implanted TNTs was higher than that of pure TNTs, and they were more conducive to cell adhesion and proliferation [133].

Additionally, Wang et al. doped carbon (C) into TNTs and found that the electrical interaction could deform the morphology of bacteria and increase the level of ROS in cells, while not compromising osteoblast growth [134].

Hence, current doping strategies can be categorized into metal doping and nonmetal doping. Metal doping, including Ag, Cu, Zn, etc., primarily takes advantage of the characteristics of broad-spectrum antibacterial activity and a low possibility of producing drug-resistant bacterial strains. Additionally, the photogenerated electrons produced by some noble metals under visible or UV light irradiation can significantly reduce the recombination probability of electrons and holes, thereby improving photocatalytic efficiency [135]. However, a higher metal concentration does not necessarily result in better antibacterial performance of the implant. On the contrary, a high doping concentration increases the cytotoxicity of implants and could be harmful to the human body.

Nonmetal dopants predominantly include elements like boron (B), N, C, sulfur (S), and other oxygen (O)-adjacent elements [121]. Following doping, alterations in implant hydrophilicity occur, along with enhancements in biocompatibility and antibacterial properties.

Doping with a single element can significantly improve the antibacterial performance of implants. However, clinical demands are diverse and extend beyond antibacterial properties. Therefore, in chemical antibacterial strategies, the focus can shift from doping with a single element to multiple elements, aiming to enhance not only antibacterial activity but also osteogenic activity and cell compatibility.

### 3.3. Drug Delivery Antibacterial Strategy

In addition to natural antibacterial metals and nonmetals, researchers have also focused on drugs with excellent bactericidal abilities to further improve antibacterial properties. Some drugs can change the structure of the surface facial mask and denature such substances as deoxyribonucleic acid (DNA), ribonucleic acid (RNA) and protein [136,137]. Unlike columnar or other solid structures, the inner part of TNTs is hollow, endowing TNTAs with excellent drug delivery and release capabilities [48]. Researchers have studied the correlation between antibacterial ability and TNT diameter, length, and drug type [47,48,138]. It has been proven that TNTAs with different TNT sizes exhibit varying drug release capabilities [139]. Specifically, longer TNT lengths provide larger inner spaces, allowing for the delivery of more drugs and effectively achieving prolonged release times [140]. In terms of TNT diameter, larger diameters facilitate drug release, while smaller diameters may hinder complete drug release [141]. However, to meet the requirements of different application environments, researchers have prepared TNTs with varying lengths, diameters, aspect ratios, and overall volumes [47]. Interestingly, it was found that changing the aspect ratio had the most significant effect on drug activity after delivery, compared to changing the other three parameters [142].

**Figure 10:**
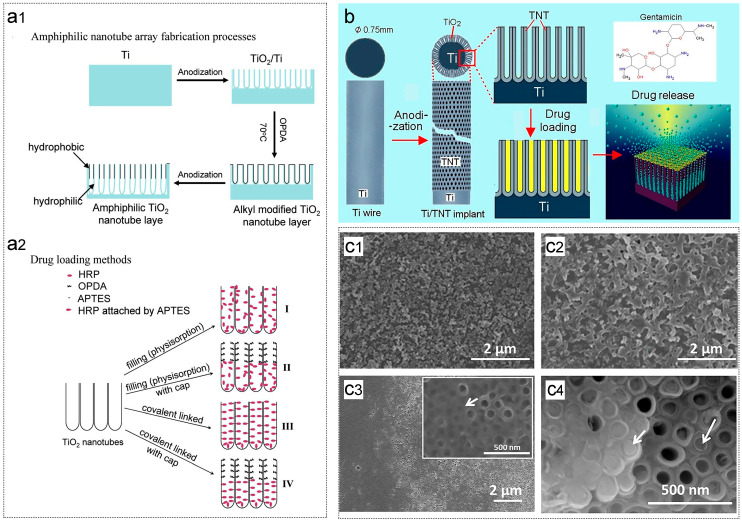
(**a1**) Scheme illustrating the procedure for fabricating amphiphilic TNTAs [143]; (**a2**) Four methods for drug loading using horseradish peroxidase (HRP) as a hydrophilic model drug: (I) Immersion without any TiO_2_ surface modification (for reference), (II) Immersion after octadecylphosphonic acid (OPDA) modification in the upper TNT layer (hydrophobic cap), (III) Covalently attached HRP over the entire TNT layers (APTES: (3-aminopropyl) triethoxysilane), (IV) OPDA cap in the upper TNT layer and covalently attached HRP in the lower TNT layer [143]; (**b**) Scheme depicting TNTs fabricated on Ti wire as a bone implant [144]; (**c1**) Vancomycin-hydroxyapatite (HA) coating on the Ti substrate [145]; (**c2**) Vancomycin-HA/collagen coating on the Ti substrate [145]; (**c3**) Areas where the TNT tops are uncovered and covered (inset, higher magnification) [145]; and (**c4**) Cracked off layer close to the top showing the presence of deposits in small amounts [145]. Reproduced with permission from Refs. [143,144,145].

Over the years, researchers have extensively investigated the unique properties of TNTs. For instance, in 2009, Song et al. proposed the fabrication of amphoteric TNTAs using a two-step electrochemical anodization process combined with hydrophobic monolayer modification (Figure 10a1) [143]. These TNTAs were envisioned as carriers for biological cells and capable of controlling drug release (Figure 10a2), thus serving as a controllable drug delivery system [143].

Similarly, Gulati et al. loaded gentamicin onto titanium wires after electrochemical anodization (Figure 10b) [144]. Their experiment demonstrated the ability to control and adjust local drug concentrations by manipulating the size (diameter and length) of the TNT structure, facilitating adaptation to the optimal treatment window for antibacterial management of bone infections [144].

In another study, Ionta et al. separately fabricated hydroxyapatite (HA) and TNTAs on titanium surfaces for carrying vancomycin (Figure 10c1–c4) [145]. Their findings revealed that HA with vancomycin exhibited faster release, whereas TNTAs prolonged release duration [145]. Both methods ensured effective antibacterial effects [145].

In addition to assessing the drug release capabilities of TNTs, researchers have investigated the antibacterial efficacy of various macromolecules and antibiotics loaded into TNTs. Yang et al. conducted electrochemical anodization of titanium plates, followed by loading quaternized chitosan into the formed TNTs [146]. Through comprehensive experiments, they concluded that titanium implants loaded with drugs on TNTs exhibit significant anti-infective potential and demonstrate favorable bone activity in vitro [146].

Furthermore, Yang et al. loaded gentamicin using freeze-drying and vacuum drying methods after electrochemical anodization of titanium [147]. Using male rats to establish an infection model, the researchers observed significant bacterial inhibition in the drug-loaded TNTA group compared to the Ti and TNTA groups [147]. These studies collectively demonstrate the feasibility of implants loaded with antibiotics via TNTs in preventing orthopedic implant-related infections.

Certainly, the early experiments validating the carrying capacity of TNTs on implants laid the groundwork for subsequent studies exploring the alteration of TNT morphology and loading various drugs. Different drugs exhibit distinct optimal concentrations and antibacterial activities against different bacteria. Moreover, the method of drug loading can significantly influence the final antibacterial performance. Therefore, continued research on drug delivery remains imperative. Below is Table 3 illustrating the loading modes and effects of some commonly used metals and drugs:

### 3.4. Combined Antibacterial Strategy

Indeed, biofilm formation leading to bacterial infections impose significant physiological distress and economic burdens on patients. Although a single antibacterial strategy also has relatively good antibacterial ability, researchers prefer to employ multiple antibacterial strategies to ensure that the implant surface does not lose its antibacterial function in the complex in vivo environment. For instance, studies have reported that the topographical antibacterial effect of TNTAs is approximately 30%, which alone may not suffice to inhibit bacterial growth and prevent infection [47]. Hence, it becomes imperative to integrate additional antibacterial strategies during the preparation of implant surfaces.

#### 3.4.1. Enhancement of Antibacterial Properties

In the preparation process of implant surfaces with TNTAs, achieving optimal antibacterial efficacy presents a delicate balance. Low concentrations of doped metal ions may result in insufficient antibacterial activity, while excessively high concentrations may lead to cytotoxicity. This principle holds true for drug loading as well. Moreover, implants loaded with a single drug offer a singular antibacterial effect, which may not adequately fulfill comprehensive clinical requirements. Consequently, researchers have explored the integration of additional antibacterial strategies, such as ion doping and drug loading, to enhance the antibacterial capability of implants.

**Figure 11:**
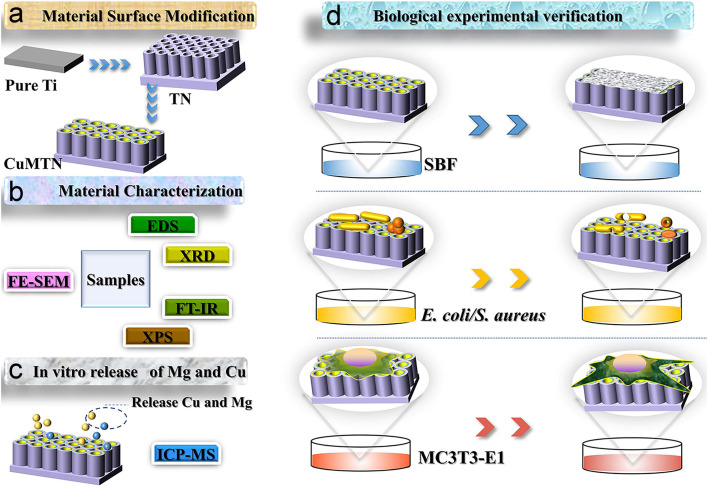
Schematic of in vitro experimental design and procedures. (**a**) Surface modification of materials to obtain metal ion-doped TNT; (**b**) Comprehensive material characterization; (**c**) Measurement of Mg and Cu release behavior in vitro; and (**d**) Biological experimental verification (including: Evaluation of in vitro biological activity using simulated body fluid (SBF); Assessment of antibacterial properties against *E. coli* and *S. aureus*; and Culture of MC3T3-E1 cells for observation of cell morphology, cytotoxicity testing, evaluation of cell proliferation, and assessment of cell differentiation). Reproduced with permission from Ref. [158].

In the realm of ion doping, Wang et al. successfully fabricated magnesium (Mg) and Cu co-doped TNTAs (CuMTNTAs) through a combination of electrochemical anodization and hydrothermal methods, as illustrated in Figure 11a–d [158]. Specifically, the surface morphology of TNTs transformed into nanoneedles following hydrothermal treatment [158]. Compared to untreated TNTs, CuMTNTs exhibited unchanged roughness but demonstrated significant enhancement in hydrophilicity [158]. Notably, CuMTNTs exhibited exceptional antibacterial properties, with antibacterial rates against *E. coli* and *S. aureus* exceeding 93% [158]. Furthermore, they promoted the proliferation and differentiation of osteoblasts, thereby endowing the implant surface with hydrophilicity, antibacterial activity, and osteogenic properties, aligning with clinical requirements [158].

Similarly, Yu et al. utilized a two-step electrochemical method to modify titanium surfaces [159]. In the first step, TNTs were formed via electrochemical anodization, followed by immersion of TNTs into AgNO_3_ solution for Ag loading [159]. Subsequently, the second step involved the production of a porous calcium–phosphorous–silver (Ca–P–Ag) surface via microarc oxidation (MAO) [159]. The average bactericidal rates against *S. aureus* for 1 and 7 days were 99.53% and 89.27%, respectively [159]. These antibacterial experiments underscored the sample’s excellent antibacterial ability, which was sustained over an extended period without exhibiting cytotoxicity [159].

Combining drug loading with ion doping represents a synergistic strategy to enhance antibacterial efficacy. Aunon et al. implemented a two-step voltage during the electrochemical anodization process to fabricate F-doped TNTs. Subsequently, a mixture of gentamicin and vancomycin was loaded onto the TNTs to prepare samples [160]. Utilizing an in vivo rabbit model, the researchers verified the biocompatibility and antibacterial properties of these samples [160]. The experiments demonstrated the ability of the samples to prevent infections caused by *S. aureus*, exhibiting excellent local bactericidal performance alongside good biocompatibility and osteosynthesis [160].

Similarly, Wang et al. initiated their process by electrochemically anodizing titanium to produce TNTs [161]. These TNTs were then modified with naringin (NAR)-loaded zeolite imidazolate framework-8, culminating in the synthesis of samples through a hydrothermal treatment [161]. These samples were capable of releasing Zn^2+^ and NAR, thereby exhibiting potent antibacterial effects against *E. coli* and *S. aureus*. In vivo experiments further corroborated the efficacy of the samples in antibacterial performance and bone integration, positioning them as promising candidates for ideal implant materials [161].

The fundamental concept of this approach revolves around leveraging the nanotube structure and electrochemical anodization technique to enhance the antibacterial efficacy of titanium-based implants through doping/loading various substances with diverse functionalities. The results demonstrate a notable enhancement in effectiveness. In comparison to samples prepared using a singular approach, those prepared via this method exhibit a more potent antibacterial effect. Consequently, our next endeavor entails appropriately pairing different substances using this method to further enhance implant performance.

#### 3.4.2. Reduction of Drug Release Rate

Currently, reducing the drug release rate, extending drug release duration, and achieving long-term antibacterial efficacy constitute a burgeoning area of interest. Research in this domain can be categorized into two distinct strategies. The first strategy involves engineering TNTs with specific geometric structures by manipulating process parameters to extend drug release duration. The second strategy entails regulating the drug release rate by integrating the drug carrier with a functional coating.

**Figure 12:**
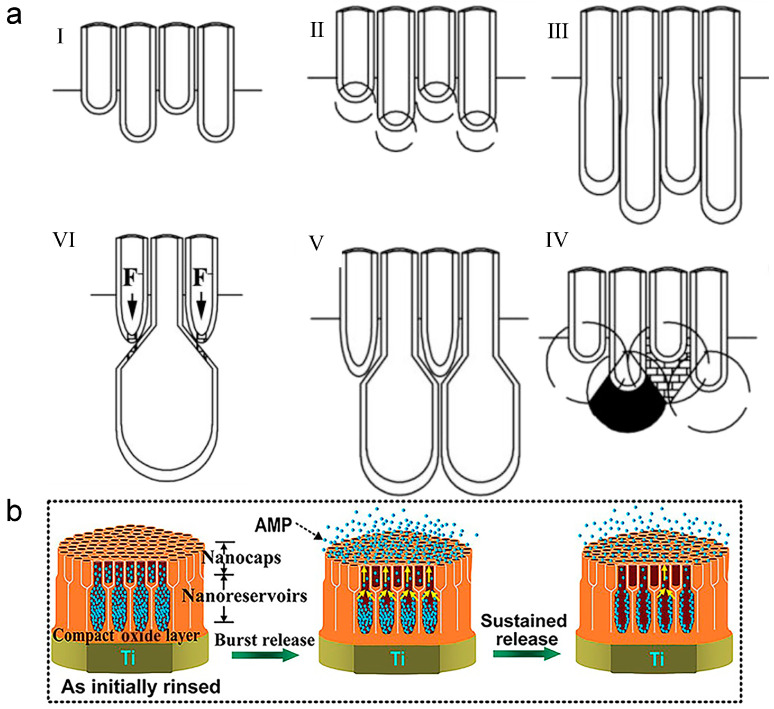
Schematic diagram of unique TNT structures. (**a**) Two-layer pear-shaped TNTs [162]. The preparation process involves two stages of electrochemical anodization [162]: (I) In the first stage, a layer of tubes is generated at a low voltage; (II, III) In the second stage, the initial voltage remains low, allowing the second layer of tubes to grow under the guidance of the first layer; (IV) As the voltage gradually increases, the adjacent tubes in the second layer begin to overlap at their midsections; (V) Over time, this overlapping progresses, resulting in the structure shown in Figure 12aV; (VI) When the voltage becomes excessively high, a large number of fluoride ions (F) diffuse, leading to the perforation of the first layer’s tube bottom and the pear-shaped tube neck; and (**b**) Double-diameter structure [163]. Reproduced with permission from Refs. [162,163].

In the first strategy, researchers encountered a bottleneck when attempting to simply adjust the diameters or lengths of TNTs to control drug release rates [39]. Consequently, they began exploring more intricate geometric structures for TNTs. In 2015, Shi et al. utilized an in situ voltage-up electrochemical anodization process to fabricate pear-shaped TNTs [162]. This process involved preparing the upper part with a small diameter at a low voltage in the first stage, followed by the preparation of the lower part with a large diameter at a high voltage in the second stage [162]. Ultimately, they successfully generated two-layer pear-shaped TNTs (Figure 12a) [162]. Compared to original TNTs, the pear-shaped structure reduced burst drug release by 50%, prolonged drug release duration, and mitigated toxicity [162].

Similar to the pear-shaped structure, Zhang et al. developed TNTAs with a double-diameter configuration (Figure 12b), followed by loading antibacterial peptides into them [163]. Within the nanostructures, the small-diameter TNTs are situated in the upper layer, serving as nano-caps to regulate drug release rates, while the large-diameter TNTs in the lower layer serve as drug reservoirs [163]. Consequently, the double-diameter structure can concurrently meet the requirements for high drug loading capacity and prolonged drug release duration [163]. In single-diameter TNTs, the antibacterial peptides were nearly fully released by the 42nd day, whereas in the double-diameter samples, peptide release could be sustained for at least 60 days [163]. Furthermore, enhancing the continuous effectiveness of the double-diameter structure could be achieved by further reducing the diameters of the TNTs in the upper layer [163]. Thanks to the unique geometric structure and properties of antibacterial peptides, this method exhibits both short-term and long-term antibacterial activity against planktonic bacteria and adherent bacteria [163].

Furthermore, Gulati et al. utilized oscillatory voltage to induce a periodic internal structure during electrochemical anodization [164]. This resulting structure effectively prevents drug loss during the washing process and diminishes the drug release rate, thereby augmenting long-term antibacterial efficacy [164].

Indeed, besides controlling the TNT structure, combining TNT with functional coatings, primarily polymers, presents a viable approach for achieving zero-order release kinetics, wherein drugs are eliminated from the body at a constant rate, irrespective of plasma drug concentration [39]. These coatings serve to reduce drug exposure by covering both the surface and openings of TNT, thereby prolonging drug release duration and enhancing antibacterial performance [39]. Certain coatings, such as polymethacrylic acid, can additionally form hydrogen bonds with drugs, further extending drug release duration [145,165].

He et al. loaded vancomycin into prepared TNTs via freeze-drying, subsequently fixing dopamine and hyaluronic acid onto the surface of vancomycin-loaded TNTs using alternate deposition technology [166]. The drug release studies demonstrated improved drug loading and controlled release within 7 days [166]. Moreover, in vitro antibacterial experiments revealed that samples prepared via alternate deposition exhibited superior antibacterial activity compared to samples loaded solely with vancomycin [166]. Thus, He et al. confirmed that coating the surface of TNTA can mitigate the limitations of TNTA by enhancing drug loading, antibacterial efficacy, and osteogenic potential [166].

Zhang et al. created micro/nanostructures on titanium surface through double acid treatment and electrochemical anodization, followed by coating Gelatin Methacryloyl (GelMA) hydrogel loaded with GKIIKLKASLKLL-NH2 (GL13K; it is a short antimicrobial peptide derived from the parotid secretory protein, and it can disrupt bacterial membranes through partial micellization and transient pore formation.) onto the micro/nanostructured titanium [167]. It was demonstrated that GL13K-releasing hydrogel-modified micro/nanostructured titanium exhibited good biocompatibility and promoted osteoblast differentiation [167]. Additionally, the sample effectively inhibited the growth of both *S. aureus* (Gram-positive) and *E. coli* (Gram-negative) by releasing GL13K [167].

**Figure 13:**
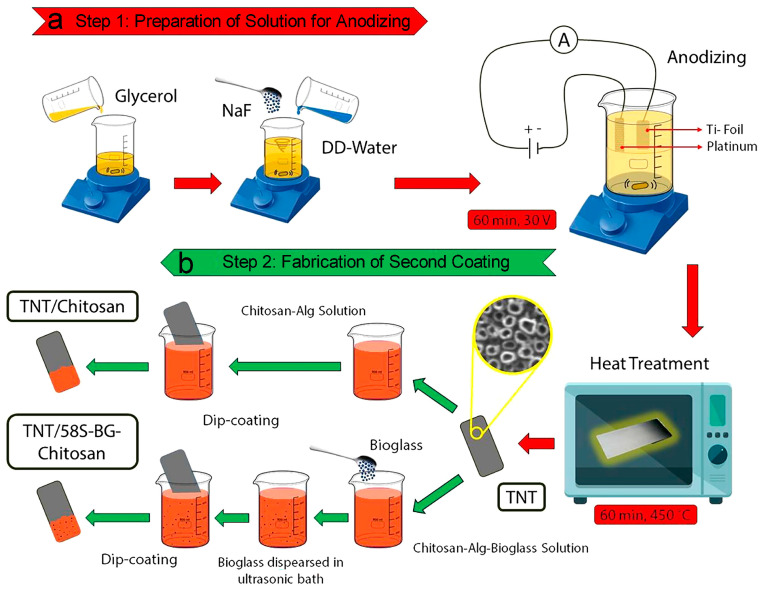
Schematic diagram illustrating the fabrication process of hybrid coatings in two steps. (**a**) Step 1: Preparation of the anodizing solution; and (**b**) Step 2: Fabrication of the second coating. Reproduced with permission from Ref. [171].

Hossein et al. fabricated TNTA and loaded gentamicin into TNTA through physical adsorption and cyclic loading [168]. They then applied layer-by-layer assembly to coat sodium alginate and chitosan onto the surface of TNTA implants in different orders [168]. The drug release results indicated that the surface coating extended the release period by approximately four weeks [168]. In addition, various other covering materials have been adopted by researchers. For instance, Ouyang et al. utilized biodegradable polymers (chitosan and polyethylene glycol) to encapsulate drug-loaded TNTA [169], Feng et al. employed a mixture film of gentamicin sulfate and chitosan [170], and Mokhtari et al. utilized chitosan and chitosan-58S bioactive glass coating, as depicted in Figure 13a,b [171].

The thickness of the coating is a crucial factor influencing drug release duration, in addition to the composition of the coating. Asadi et al. coated different layers of chitosan onto TNTs loaded with ciprofloxacin to achieve a continuous release profile [172]. Their in vitro drug release study demonstrated that drug release persistence increased with the number of chitosan coating layers [172]. Similarly, Wang et al. modified the drug release curve by varying the thickness of the polymer film on the surface of TNTs to optimize therapeutic efficacy throughout the treatment period [173]. Adjusting the polymer coating thickness appropriately significantly improved drug release characteristics, extending total drug release time from 5 days to over 40 days [173].

However, it is essential to note that the thickness of the coating on the TNT surface does not follow the principle of “the thicker, the better.” Sun et al. coated the TNT surface with polylactic-co-glycolic acid (PLGA) films of varying thicknesses: 50 nm, 250 nm, and 800 nm [174]. Their findings revealed that TNTs with 50 nm and 250 nm thick films provided an initial short pulse of drug release, followed by smooth and continuous release of drugs over several weeks [174]. Conversely, the 800 nm thick film completely blocked drug release in the first 4 weeks, resulting in drug concentrations too low to achieve effective sterilization [174].

Both altering the shape of TNTA and applying special coatings to TNTA can achieve the goal of reducing the drug release rate. Therefore, future research should focus on developing more complex shapes that enable longer release times. Alternatively, researchers can explore coatings with specific functions to extend release time or provide additional biological properties while meeting antibacterial requirements.

## 4. Conclusions and Future Perspectives

With the advancement of technology and increasing healthcare needs, implant surgeries have seen a steady rise over the past decades. However, relatively high post-surgery infection rates have underscored the urgent need for improved antibacterial performance of implant surfaces. Titanium dioxide, renowned for its non-toxicity, excellent biocompatibility, and corrosion resistance, stands out as a widely used biomaterial. The highly ordered TNTA has garnered significant attention due to its micro/nanostructure, which inhibits bacterial reproduction and exhibits photocatalytic antibacterial activity. Despite these advantages, TNTA still lacks sufficient antibacterial efficacy against various infection-related bacterial strains. Therefore, it is imperative to explore suitable antibacterial strategies to endow TNTA-coated implant surfaces with excellent short-term and long-term antibacterial properties, meeting the demands of clinical applications.

In this review, we commence by introducing various preparation methods for TNTA, highlighting the advantages of the electrochemical anodization method. Subsequently, we delve into the evolution of the electrochemical anodization method, categorizing it into four stages based on different electrolytes. We elucidate the chemical mechanism of electrochemical anodization and the formation process of TNTs. Additionally, we discuss the impact of different electrochemical anodization parameters on the morphology and geometric dimensions of TNTs.

Regarding antibacterial strategies, we categorize them into four main categories: topographical, chemical, drug delivery, and combined antibacterial strategies. While the topographical antibacterial strategy can achieve long-term antibacterial effects, its efficacy alone may not suffice to inhibit post-surgery infections adequately. Consequently, researchers have turned to incorporating chemical or drug delivery antibacterial strategies to overcome this limitation.

Chemical ion doping represents a method to enhance the photocatalytic performance of TNT and confer antibacterial effects through the release of metal or non-metal elements. However, it is crucial to note that doping appropriate elements at optimal concentrations is paramount for avoiding shortcomings in antibacterial efficacy or the emergence of cytotoxicity.

Likewise, drug delivery mechanisms can achieve antibacterial effects by releasing drugs in a controlled manner. The quantity and release rate of drugs play a pivotal role in determining the antibacterial performance of TNT. Excessively high drug concentrations can pose harm to healthy cells and tissues. Consequently, selecting the appropriate type of drug, optimizing the drug loading method, and striking a balance between drug delivery capacity and release rate are pressing challenges that require attention.

In pursuit of achieving satisfactory antibacterial capabilities for clinical applications, researchers are exploring the combination of various antibacterial strategies. These include techniques such as co-doping with multiple elements and drug loading into TNTs with specialized geometric structures. This article provides a comprehensive review of the current advancements in combined antibacterial strategies and summarizes their distinctive characteristics.

Looking ahead, it is anticipated that future research efforts will prioritize the comprehensive enhancement of the biological characteristics of implants, encompassing antibacterial performance, cell compatibility, and osteogenic activity, among others, to fulfill the diverse requirements in clinical applications. However, achieving these biological characteristics solely through a single strategy poses challenges. Therefore, there is a pressing need to systematically investigate combined antibacterial strategies in the future.

## Figures and Tables

**Table 3:** Loading modes and effects of common elements and drugs.

		Loading Method	Effect	Ref.
Doped Elements	Au	Magnetron-sputtered	Antibacterial ability	[148]
	Ag	Immersing in AgNO_3_ solutions	Antibacterial ability	[127]
	Cu	Electrochemically anodization	Antibacterial ability and angiogenesis	[149,150]
	Zn	Polydopamine chelation	Antibacterial ability and osteogenesis	[151]
	Sr	Hydrothermal treatment	Osteogenesis	[152]
	F	Plasma treatment	Antibacterial ability and osteogenesis	[153]
Drugs	Vancomycin	Physical absorption	Antibacterial ability	[154]
	Gentamicin	Immersion	Antibacterial ability	[155]
	Tetracycline	Lyophilization method, vacuum drying	Antibacterial ability	[156]
	Cecropin B	Lyophilization	Antibacterial ability	[157]

## Data Availability

The original contributions presented in the study are included in the article, further inquiries can be directed to the corresponding authors.
